# An Analysis of Vegetation and Microbiome Recovery in Abandoned Agricultural Fields

**DOI:** 10.1002/ece3.72865

**Published:** 2026-01-05

**Authors:** Heike Oosthuysen, Kayleigh Coetzer, M. Thabang Madisha, Willem G. Coetzer

**Affiliations:** ^1^ Department of Genetics University of the Free State Bloemfontein Free State South Africa; ^2^ Leliekloof Farm Cradock Eastern Cape South Africa; ^3^ Department of Zoology and Entomology University of Fort Hare Eastern Cape South Africa

**Keywords:** 16S rRNA, bacterial community, biodiversity, high‐throughput sequencing, plant community, restoration, soil health

## Abstract

Biodiversity is a key indicator of an ecosystem's resilience to disturbances, but farming practices like monocultures can be disruptive. Assessing the biodiversity levels in abandoned fields can help to reveal recovery patterns and inform strategies to conserve biodiversity in agricultural landscapes. The main aim of this study was to assess the pace of natural recovery for a chronosequence of formerly planted fields in a grassland habitat in the Eastern Cape, South Africa. The plant communities were evaluated using species counts, while the bacterial communities were assessed through high‐throughput sequencing (HTS) of the 16S rRNA gene. The alpha diversity indices indicated that the diversity levels within the old fields have started to resemble natural conditions for both the plant and microbial communities. Furthermore, the NMDS analyses identified clear variations in bacterial and plant community compositions among differently aged successional groups and the natural habitats. This study provides evidence that biodiversity levels within crop fields can recover from agricultural disturbances. However, considering the significant changes in climate and rainfall patterns in the study area, it remains unclear whether the community structures of the crop fields will reach native conditions in the coming decades, if at all.

## Graphical abstract

This study investigated the natural recovery of biodiversity in formerly cultivated fields in the Eastern Cape, South Africa, by comparing plant and bacterial communities across different successional ages. We found that diversity levels in old fields began to resemble natural conditions for both community types, indicating a positive recovery trajectory from agricultural disturbances. However, due to significant regional climate shifts, it is uncertain whether these communities will fully return to their native state.

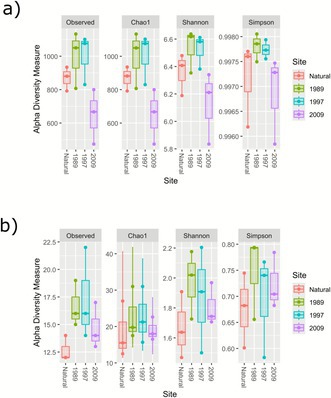

## 1 Introduction

Soil forms a complex system consisting of various microhabitats that fluctuate in composition depending on the environmental conditions that shape them (Tiedje et al. 2009). Land‐use systems further influence the fertility and stability of an ecosystem (Karki et al. 2022). Good soil health is vital to the overall ecosystem as it ensures plant life's survival, which in turn drives plant and animal diversity (Bargali et al. 2019). Soil health can be broadly defined as the capacity of soil, including its living components like microorganisms, to function within both natural and human‐altered ecosystems, supporting and sustaining plant and animal productivity and health (Doran 2002). Most of the soil microorganisms have been reported to improve soil fertility and increase plant growth, play critical roles in organic matter decomposition, nutrient recycling and ecosystem stabilisation, which are vital components of any ecosystem (Torsvik and Øvreås 2002; Nabi 2023). Natural stocks of plant nutrients exist in soils but are mainly inaccessible to plants, with only small percentages of these nutrients released each year by biological or chemical processes (Swami 2020). However, this release is too slow to compensate for nutrient losses, especially when these losses are accelerated by agricultural output. Microorganisms, therefore, fulfil a key function in converting organic matter within soils into simpler, inorganic forms readily available for plant absorption (Magdoff and van Es 2021). Microbiome activity not only boosts a plant's performance by supporting soil fertility but can also increase plant tolerance to abiotic challenges, including changes in water availability or extreme temperatures (Lau and Lennon 2012; Pereira 2016), as well as biotic challenges like diseases, herbivore prevalence and habitat disturbance (Harun‐Or‐Rashid and Chung 2017; Bell et al. 2019; Dove et al. 2021).

The quantity and quality of soil organic matter, pH and redox potential conditions all influence the physical and chemical attributes of the soil. These factors also significantly impact the dynamics and structure of the various microbial, animal and plant communities in terrestrial environments (Ramírez‐Flandes et al. 2019; Kang et al. 2021). The nutrient content of soil is influenced by the available amendments of litter/organic manure and serves as one of the inputs of nutrients in ecosystems (Pant et al. 2017; Padalia et al. 2022). The soil nutrient depletion and degradation of soil health due to various agricultural cropping systems are globally well studied (Trivedi et al. 2016; Hartmann and Six 2023). Several studies have also shown how the recovery of old crop fields can occur at different rates, and the extent of recovery varies between habitats (Morris et al. 2011; Krause et al. 2016; Coetzer and Coetzer 2023). Understanding, at a local scale, how the microbiome and soil properties are altered by disturbances and the recovery processes following these disruptions can provide valuable insights into the complex relationships between the soil microorganisms and vegetation communities during succession. Coetzer and Coetzer (2023) reported on the changes in soil quality between old crop fields (abandoned 12–32 years at the time of sampling) and the surrounding natural veld at the same study site as the current study. Their results indicated no significant distinctions between the natural sites and the old crop fields when comparing soil density, pH, salinity (TDS) and soil texture. Soil water holding capacity (WHC), carbon % (C %), nitrogen % (N %), carbon stock (C stock) and nitrogen stock (N stock) did, however, exhibit significant disparities between the natural sites and the old crop fields. These findings suggest that the old fields are reverting to natural conditions, although not entirely. While studying the effect of land‐use changes on the diversity and community composition of the soil microbial communities in African grassland soils, Nkuekam et al. (2018) showed that agricultural soils contain distinct bacterial communities compared with natural grassland soils. The dissimilarities observed in microbial communities were attributed to variations in soil chemical properties. These findings support the concept that soil chemical properties, as well as the compositions and diversities of microbial communities, undergo alterations following the conversion from a natural to an agricultural system.

The global changes in weather and rainfall patterns have significantly fostered a global increase in natural recovery processes, including passive techniques such as cropland abandonment (Shackleton et al. 2013; Blair et al. 2018; Cava et al. 2018). Cropland abandonment refers to a passive restoration method where croplands are removed from active agricultural production without future plans for cultivation (Baxter and Calvert 2017). There are many advantages associated with cropland abandonment, including but not limited to the restoration of ecosystem services (Tallis et al. 2008), the improvement of soil quality (Chang et al. 2017), increased biodiversity levels (San Roman Sanz et al. 2013; García‐Llamas et al. 2019) and reductions in soil erosion (García‐Ruiz and Lana‐Renault 2011). However, there are also disadvantages to cropland abandonment. A review by Rey Benayas et al. (2007) identified five main problems associated with cropland abandonment, namely, reductions in landscape heterogeneity that lead to vegetation homogenisation, desertification, reduction of water stock, reductions in populations of adapted species resulting in biodiversity loss, and lastly the loss of cultural and aesthetic land value.

Restoration projects vary in their objectives and the approaches used to accomplish them. Restoration success is frequently measured in terms of the recovery of structural elements of the disrupted ecosystem, including species composition, diversity, density and cover, as well as stand structure, which varies depending on the amount of time since restoration began, the type of disturbance and the landscape context (Crouzeilles et al. 2016). The restoration of plant communities following disturbances, such as agricultural activities, unfolds in a distinct sequence. Specific species are known to initially colonise the disturbed area, later replaced with more specialised species as soil conditions improve. van Oudtshoorn (2020) described three vegetation succession stages in Southern African grasslands that occur after a disturbance. Starting with the pioneer stage, hardened, fast‐growing pioneer plants that can grow in adverse conditions will colonise the disturbed area. The subclimax stage follows once the pioneer species have partially or entirely transformed the environment. During this stage, subclimax plants, which form denser growths than pioneer plants and offer more protection to the soil, are established. The subclimax stage is often characterised by a dynamic and changing plant community, with different species dominating at different times or microhabitats. Finally, the climax stage is characterised by strong perennial plants well adapted to the improved environmental conditions (van Oudtshoorn 2020).

Agricultural intensification is one of the primary factors contributing to the conversion and degradation of the grassland biome within South Africa. Studies looking at the ecological consequences and subsequent management options of abandoned croplands in developing countries, such as South Africa, are relatively limited when compared with the extensive research done in, for example, Western Europe (Verburg and Overmars 2009; Pereira and Navarro 2015). The studies that have been done on cropland abandonment in South Africa have reported varying rates of recovery between sites (van der Merwe and van Rooyen 2011; Blair et al. 2018; Shackleton et al. 2019; Sibiya et al. 2023). The inconsistency in the results can be attributed to variations in research design and whether the research focused on the pre‐ or post‐abandonment period of the fields (Queiroz et al. 2014).

The main aim of the current study was to assess the rate of natural recovery of the biotic elements for a chronosequence of formerly planted fields in a grassland habitat in the Winterberg mountain range in South Africa. The extent of recovery was evaluated by considering the vegetation and bacterial microbiome diversity levels in three old crop fields at different ages since abandonment, compared with the undisturbed natural habitats surrounding these fields. Firstly, the vegetation diversity was assessed through species counts in the respective fields using the step‐point method. Secondly, the soil microbiome was evaluated using high‐throughput sequencing (HTS) of environmental DNA extracted from soil samples, targeting the 16S rRNA gene. The results obtained from the study will provide an estimate of habitat recovery in terms of the plant and microbial species richness, diversity and community composition of the different fields.

## 2 Materials and Methods

### 2.1 Study Site

This study was conducted on a commercial livestock farm in the Winterberg Mountains near Cradock, in the Eastern Cape, South Africa (32°19′55.4″S 26°00′58.0″ E). The farm, Leliekloof, falls within the grassland biome consisting of two vegetation types: the Karoo Escarpment Grassland and the Tarkastad Montane Shrubland (Mucina and Rutherford 2006). The topography in this region ranges from flat plains to rolling hills, including mountainous regions and escarpments. The vegetation in this biome tends to be short‐lived and dominated by grasses due to the moderate to high rainfall and the high elevation of these mountainous regions. The area is also known for its abundant herbaceous plants, with several endemic taxa (Clark et al. 2014). The site is situation in the Arid, Steppe and Cold (Bsk) Köppen–Geiger climate region (Beck et al. 2018), and is geologically represented by the Adelaide subgroup of the Beaufort Group of sedimentary layers as well as Karoo Dolerite intrusions (Council for Geoscience 2021).

**FIGURE 1:**
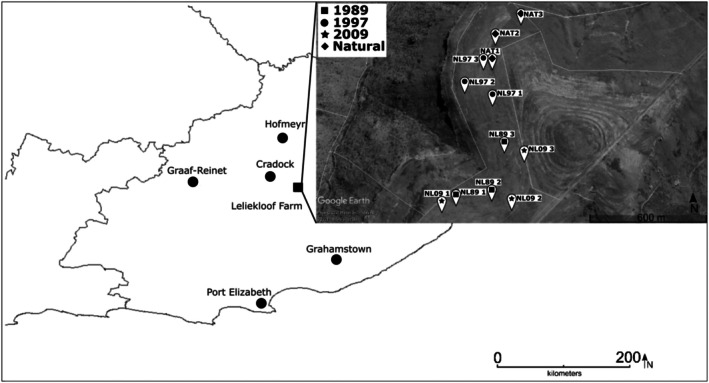
Map illustrating the location of the 12 sampling sites in the Winterberg Mountains, South Africa. This figure is a modified rendition of the map used in a parallel study by (Coetzer and Coetzer 2023). The terrain map shows the individual sample sites and was extracted from Google Earth Pro (Google Earth Pro 2021).

The sampling sites are located on a plateau at 1658 m above sea level (m.a.s.l.), with an average rainfall of 365.36 mm from April 2017—April 2021 (J.M. Coetzer, personal communication). The abandoned crop fields used in this study were once planted with wheat and oats in rotation but are now primarily used as natural grazing for livestock. Planting wheat and oats became less feasible due to a gradual change in rainfall intensity and timing in the area, which led to crop field abandonment (J.M. Coetzer, personal communication). The sampling sites include old crop fields last planted in 1989, 1997 and 2009 and the surrounding natural grassland (as reference sites), as indicated in Figure 1. The relative abundance per plant family was further estimated from the count data.

### 2.2 Sampling Methodology

The vegetation surveys and soil sample collections were conducted between 29 March and 6 April 2021, following the rainy season. A total of 12 plots were surveyed, three for each age group, as well as the natural grassland (*n* = 3 per site). Each plot was set up as a 9 m × 9m grid, with points marked 1 m apart, resulting in a total of 100 points per plot. The plant biodiversity for each plot was assessed through species counts using the step‐point method (Evans and Love 1957). Each individual plant count was determined by lowering a sampling pin, using the definite marks on the grid as a guide. When the pin hit a plant, bare ground, or a rock, an identification was made and recorded. The plant count data is depicted in Table S1.

At each plot, five soil cores (4.5 cm in diameter) were collected at a depth of 5 cm. Clean gloves were used during the sampling of each plot to ensure no cross‐contamination between plots. The soil corers were cleaned using water and sterilised using 2% sodium hypochlorite and 70% ethanol before sampling each plot. The five samples were pooled into a single sterile sealable plastic bag to ensure the soil samples were representative of each sampling plot and placed in a cooler bag with ice packs to minimise further bacterial growth. Sixty individual soil samples were collected, resulting in 12 pooled samples representing each plot.

### 2.3 DNA Extraction and HTS Data Processing

The soil DNA extraction was performed using the E.Z.N.A. Soil DNA Kit (Omega Bio‐Tek Inc., Norcross, United States) according to the manufacturer's protocol. Two negative controls (extraction blanks) were incorporated into the experimental design to account for any non‐specific effects, contamination, or background noise that may occur during the experimental process. The integrity of the DNA was checked on 1% agarose gels, and the yield and purity of the DNA were determined using a Nanodrop ND‐1000 Spectrophotometer (NanoDrop, Thermo Fisher Scientific, Waltham, MA, USA). HTS following the bTEFAP Illumina MiSeq (2 × 300 bp PE) technology, as developed by Dowd et al. (2008), was performed targeting the V1‐V3 hypervariable region of the 16S rRNA gene, using primers 27F (AGAGTTTGATCMTGGCTCAG) and 519R (GWATTACCCGCGGCKGCTG; Klindworth et al. 2012). All HTS sequencing was outsourced to MR DNA laboratories (www.mrdnalab.com, Shallowater, TX, USA) on an Illumina MiSeq instrument following the manufacturer's guidelines. DNA amplification was achieved through a 30‐cycle Polymerase Chain Reaction (PCR) using the HotStarTaq Plus Master Mix Kit (Qiagen, USA) under the following specified conditions: 94°C for 3 min, followed by 30–35 cycles of 94°C for 30 s, 53°C for 40 s and 72°C for 1 min, after which a final elongation step at 72°C for 5 min was performed. Non‐template controls were also included at each PCR reaction. The PCR products were checked in 2% agarose gel to determine the amplification success and the relative intensity of the bands. The samples were purified using Ampure XP beads, which were then used to prepare the Illumina DNA library. Only the two negative controls from the DNA extraction process were brought through to sequencing. All raw sequences are available on the NCBI GenBank database under BioProject PRJNA1043901 (Accession numbers are shown in Table 1).

**TABLE 1:** Sample and HTS information for the 12 soil samples and two control samples used in this study.

Sample ID	Age group	Reads	ASVs	Latitude	Longitude	Accession no
NL09 1	2009	72,427	1385	−32.335483	26.014450	SRS19611870
NL09 2	2009	60,641	954	−32.335617	26.017917	SRS19611871
NL09 3	2009	98,656	1591	−32.333617	26.018617	SRS19611881
NL97 1	1997	104,350	1918	−32.331000	26.017117	SRS19611893
NL97 2	1997	117,233	2030	−32.330400	26.015633	SRS19611904
NL97 3	1997	133,186	2236	−32.329350	26.016800	SRS19611909
NL89 1	1989	104,160	1840	−32.335250	26.015167	SRS19611910
NL89 2	1989	91,001	1513	−32.335133	26.016917	SRS19611911
NL89 3	1989	137,853	2298	−32.333117	26.017633	SRS19611912
NAT 1	Natural	116,401	2033	−32.329233	26.017167	SRS19611913
NAT 2	Natural	132,585	2011	−32.328033	26.017500	SRS19611875
NAT 3	Natural	120,714	2030	−32.328033	26.018883	SRS19611874
Total	—	1,289,207	21,839	—	—	
Mean	—	107,434	1820	—	—	
Control 1	Control	5547	52	—	—	SRS19611873
Control 2	Control	7710	58	—	—	SRS19611872

*Note:* The sample ID, age group, number of reads, number of observed ASVs, GPS coordinates and accession numbers are provided. The two negative control samples yielded a negligible amount of data.

The software package QIIME 2 v.2022.2 (Bolyen et al. 2019) was used to process the raw demultiplexed sequences obtained from the sequencing facility. Sequencing primers and adapters were removed using the Cutadapt plugin (Martin 2011), followed by sequence data trimming, denoising and grouping into amplicon sequence variants (ASVs) using the DADA2 plugin (Callahan et al. 2016). It was observed that the reverse reads were of low quality and negatively impacted our percentage of retained sequences following the filtering and denoising steps. It was therefore decided to only utilise the forward reads for all further analyses, providing information on the V1‐2 regions of the 16S rRNA gene. A quality threshold of 30 (Q30) was applied to refine the dataset further, ensuring that only sequences meeting the minimum quality score were retained for the subsequent analyses. Taxonomy was then assigned to the ASVs using a naive Bayes taxonomy classification algorithm against the SSU SILVA (Version 132) 99% OTUs reference sequences (Pruesse et al. 2007; Quast et al. 2013). Additionally, mitochondrial and chloroplast sequences were filtered from the dataset.

### 2.4 Statistical Analysis

Statistical analyses of the sequencing data were performed using the phyloseq (McMurdie and Holmes 2013), vegan (Oksanen et al. 2020), dplyr (Wickham et al. 2023) and DESeq2 (Love et al. 2014) packages in R statistical software (R Core Team 2021). The plant count data was also imported into R, where the same analyses as the bacterial ASV data were performed to ensure a parallel examination of both plant and bacterial datasets. Rarefaction was conducted on the bacterial sequence data to standardise sequencing depths across all samples, mitigating the impact of unequal sampling efforts and ensuring fair comparisons of microbial diversity metrics. Additionally, rarefaction curves were constructed for both datasets to assess the adequacy of the sampling efforts. The relative abundance was estimated for the bacterial and vegetation datasets at phylum and family level, with data grouped by sample sites and age since abandonment. Abundance bar plots were generated using the R phyloseq package for both datasets. Linear regression analyses were performed to analyse the trend in the top four bacterial phylum relative read abundance (RRA) as a function of successional age, as well as the trend of the top four plant genera abundances as a function of successional age. This was performed using the R stats package lm() function.

Alpha diversity was assessed by calculating the observed species richness, Chao1 (Chao 1984), the Shannon–Weiner diversity index (H) (Shannon 1948) and Simpson's diversity index (D) (Simpson 1949). The estimates were performed based on the occurrence and abundance data from the lowest taxonomic ranks of the microbial and vegetation dataset. The combination of these diversity indices was chosen as they provide valuable insights regarding the species richness, evenness and overall diversity of the respective communities. A multivariate analysis of variance (MANOVA) was then conducted to test whether the time since abandonment significantly affects the alpha diversity measures across the various age groups.

A non‐metric multidimensional scaling (NMDS) based on the Bray–Curtis dissimilarity matrix (Bray and Curtis 1957) was carried out to visualise the community structure of the four age groups in a two‐dimensional ordination space. The NMDS plots were calculated for the bacterial and plant datasets separately. Bray–Curtis was selected because of its ability to handle community matrices with many zeros, which can often cause zero‐inflation‐related issues in other dissimilarity indices. Permutational multivariate analysis of variance (PERMANOVA) with 999 permutations was used to assess the influence of environmental factors and soil parameters (sourced from Coetzer and Coetzer 2023) on the microbial and plant community structures. The environmental conditions considered include age since abandonment, percentage of bare ground observed for each site, and percentage dead vegetation at the respective sampling sites. The year 1965 was chosen as a time reference for the natural sites, as it corresponds to the initial establishment of the fields (JM Coetzer; personal communication). The soil properties assessed include N stock, N %, C stock, C %, WHC and soil pH levels as sourced from Coetzer and Coetzer (2023).

Differential abundance testing was performed to identify the bacterial families responsible for the observed differences in microbial community composition. DESeq2 uses negative binomial models and ASV (or OTU) read counts to test whether individual bacterial taxa are differentially abundant across experimental factors. In DESeq2 analysis, log_2_foldchange analysis (LFCa) is employed to quantify and express the magnitude of gene expression changes between two experimental conditions, facilitating the identification of upregulated and downregulated genes based on a logarithmic scale. Furthermore, *p*‐values are converted to *Q*‐values to correct for multiple hypothesis testing using a threshold of *Q* < 0.05 for significance (Love et al. 2014).

## 3 Results

After filtering, 1,289,207 reads (mean: 107,434) were obtained from the 12 pooled soil samples sequenced at the 16S rRNA gene (Table 1). Rarefaction curves were generated for both the bacterial and plant data, with the bacterial dataset slope plateauing, indicating that the diversity of all the samples has been fully observed. However, the plant dataset did not reach a plateau, indicating that the species richness in the studied sites has not been fully observed (Figure S1). The number of bacterial ASVs detected for the samples analysed ranged from 954 to 2298 per sample (Total: 21,839). The four sample groups shared 677 bacterial ASVs, while 68, 8, 27 and 5 were found to be unique to the Natural, 1989, 1997 and 2009 age groups, respectively (Figure S2). Vegetation species overlaps were also observed, with 8 species observed in all four age groups. Four plant species were unique to the Natural group, with 5, 0 and 3 species unique to the 1989, 1997 and 2009 age groups, respectively (Figure S3).

### 3.1 Relative Abundance

**FIGURE 2:**
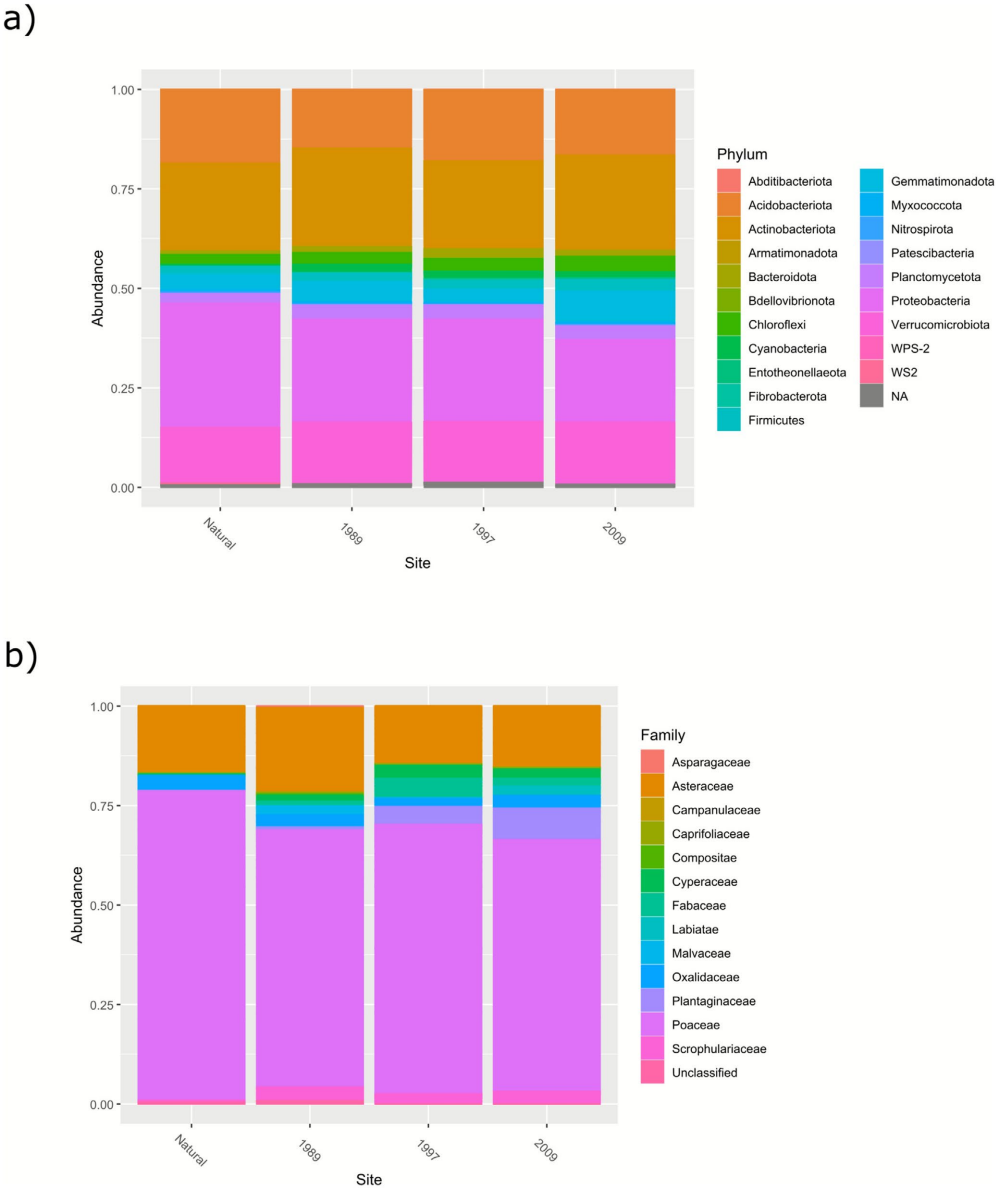
Stacked bar plots showing the relative abundance at the family level for the restoration chronosequence (Natural, 1989, 1997 and 2009) for the (a) bacterial community (> 1%), based on non‐normalised ASV counts, whereas the (b) plant community bar plot is based on species counts (colour version can be viewed online).

**FIGURE 3:**
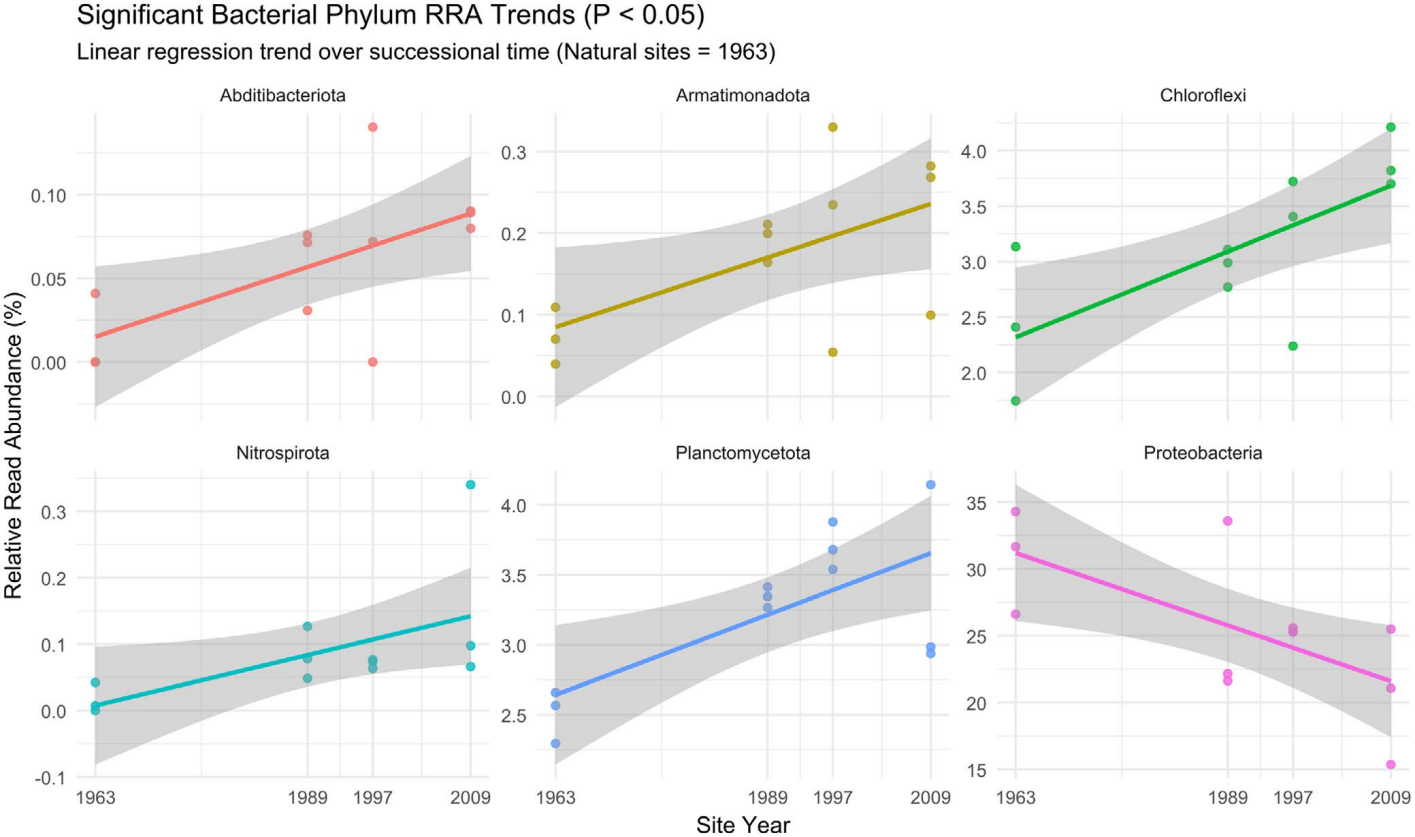
Linear regression of bacterial phylum relative read abundance (RRA) as a function of time since abandonment. The year of abandonment is on the *x*‐axis and serves as a chronosequence, with 1963 representing the natural vegetation sites and 1989–2009 representing the older to younger abandoned crop fields.

The bacterial phyla with an RRA of > 10% include Proteobacteria (26.5%), Actinobacteria (23.3%) and Verrucomicrobiota (14.8%) (Figure 2a). The bacterial phyla Proteobacteria, Actinobacteriota and Acidobacteriota were identified as the three most abundant taxa for the Natural, 1997 and 2009 groups, with Proteobacteria, Actinobacteriota and Verrucomicrobiota more abundant in the 1989 group (Table S2). Six bacterial phyla were identified with statistically significant trends (*p*‐value < 0.5) across the study sites (Figure 3). Proteobacteria was the only phylum with a significantly negative trend, decreasing in abundance chronologically from the Natural (1963) group to the 2009 group (*R* ^2^ = 0.45; *p*‐value = 0.017). Five bacterial phyla showed statistically positive trends, increasing in abundance from the Natural (1963) group to the 2009 group, and included Chloroflexi (*R* ^2^ = 0.522, *p*‐value = 0.008), Planctomycetota (*R* ^2^ = 0.489, *p*‐value = 0.011), Abditibacteriota (*R* ^2^ = 0.416, *p*‐value = 0.024), Armatimonadota (*R* ^2^ = 0.356, *p*‐value = 0.041) and Nitrospirota (*R* ^2^ = 0.346, *p*‐value = 0.044).

**FIGURE 4:**
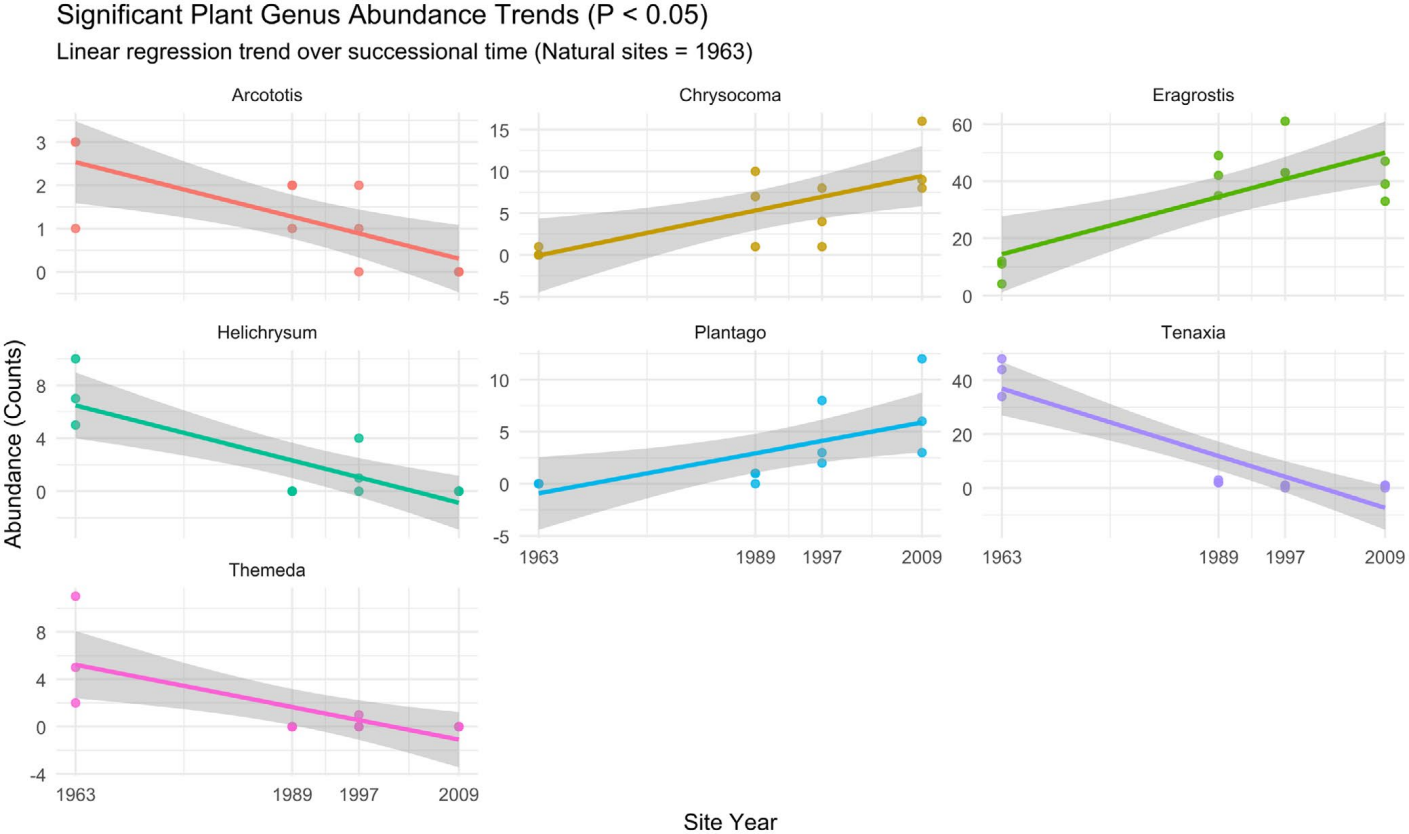
Linear regression of vegetation genera abundance (counts) as a function of time since abandonment. The year of abandonment is on the *x*‐axis and serves as a chronosequence, with 1963 representing the natural vegetation sites, and 1989–2009 representing the older to younger abandoned crop fields.

During the plant survey, one plant phylum, Magnoliophyta, was identified, with the identified plant families observed for each sample shown in Figure 2b. The plant families were dominated by Poaceae (68.20%), followed by Asteraceae (16.98%), Plantaginaceae (3.53%), Oxalidaceae (2.75%), Scrophulariaceae (2.45%), Fabaceae (2.06%) and Cyperaceae (1.96%). Within each group, Poaceae and Asteraceae dominated, with the third most abundant family differing for each group (Table S2). The grass genus *Eragrostis* was observed as the dominant plant taxon in all three old crop fields, with the grass genus *Tenaxia* dominating the natural vegetation cover (Figure S4). A high percentage of dead plant material in the form of a grass moribund layer was observed for the natural sites. Seven plant genera showed statistically significant trends (*p*‐value < 0.05) across the study sites (Figure 4). Four genera showed significant negative trends, decreasing in abundance chronologically from the Natural (1963) group to the 2009 group. These were *Tenaxia* (*R* ^2^ = 0.821, *p*‐value < 0.001), *Helichrysum* (*R* ^2^ = 0.669, *p*‐value = 0.001), *Arcototis* (*R* ^2^ = 0.563, *p*‐value = 0.005) *and Themeda* (*R* ^2^ = 0.534, *p*‐value = 0.007). Three genera showed significant positive trends, increasing in abundance chronologically from the Natural (1963) group to the 2009 group. These were *Eragrostis* (*R* ^2^ = 0.624, *p*‐value = 0.002), *Chrysocoma* (*R* ^2^ = 0.519, *p*‐value = 0.008) and *Plantago* (*R* ^2^ = 0.468, *p*‐value = 0.014).

### 3.2 Diversity and Community Structure

**FIGURE 5:**
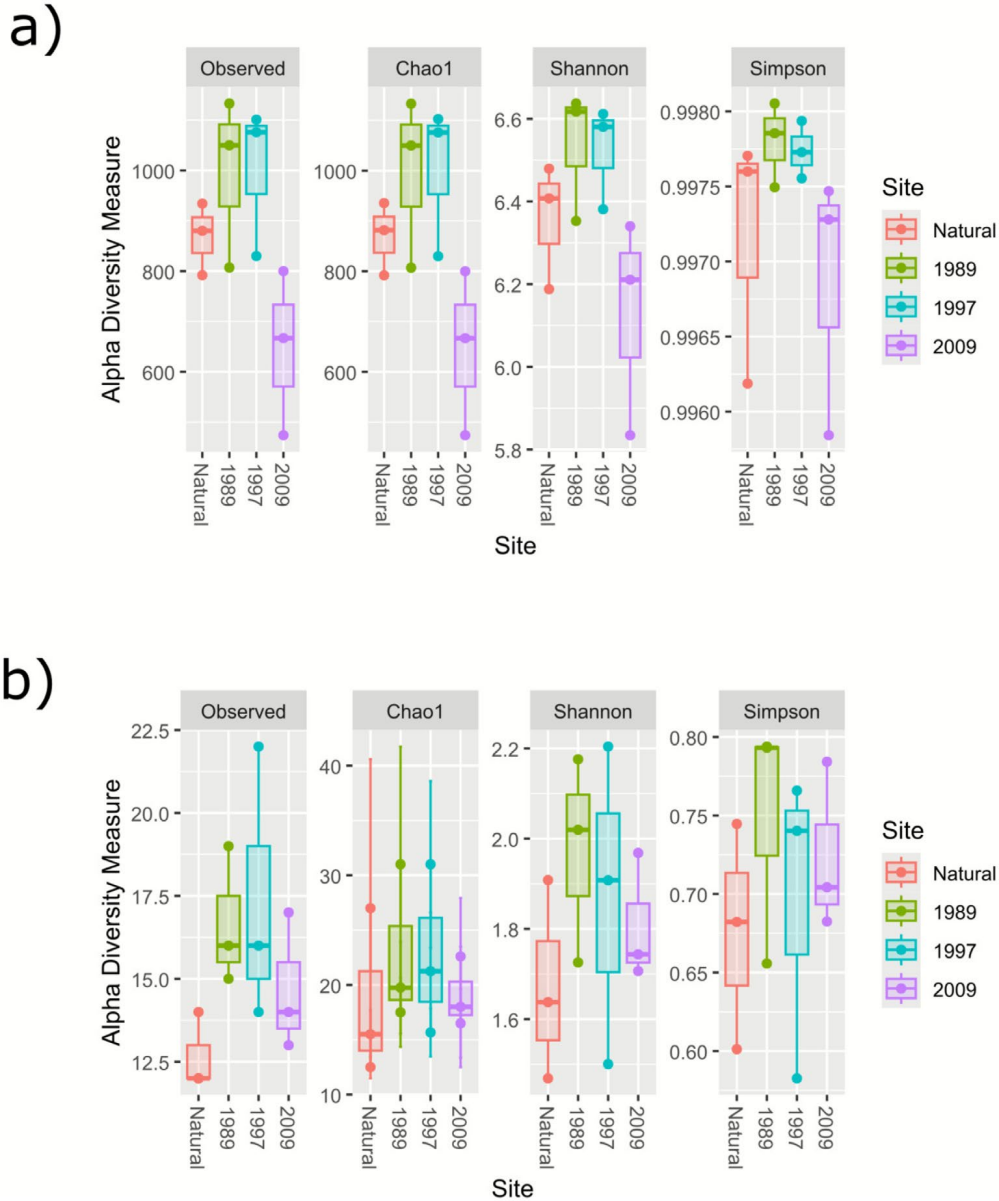
Boxplots showing the alpha diversity indices for the restoration chronosequence (Natural, 1989, 1997, and 2009). Observed species richness (S), Shannon–Weiner diversity index (H) and Simpson's diversity index (D) are shown for the (a) bacterial and (b) plant communities.

The highest mean alpha diversity values for the bacterial dataset were observed for the 1997 and 1989 sample groups (Figure 5a), with the lowest values observed in the 2009 group. For the plant community (Figure 5b), the highest mean alpha diversity values were observed in the 1989 and 1997 groups, with the lowest values observed in the natural sites. The MANOVA results showed, however, that the alpha diversities of the groups are generally not affected by age in a statistically significant manner (*p* > 0.05).

**FIGURE 6:**
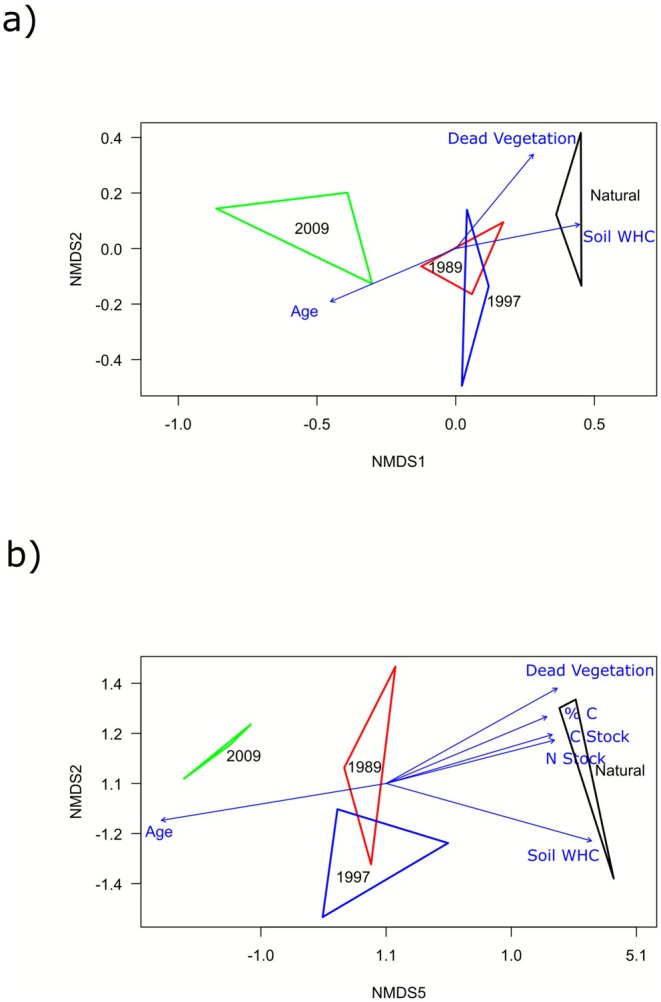
Non‐metric multidimensional scaling (NMDS) based on the Bray–Curtis dissimilarity matrix visualising the relative differences for the (a) bacterial and (b) plant communities. The environmental factors with a significant effect (*p* < 0.05) on the communities are indicated in blue.

The NMDS results based on the Bray–Curtis diversity index visualised the dissimilarity of the bacterial (Figure 6a) and the plant communities (Figure 6b). For both communities, the samples collected from each group clustered together, suggesting similarity within each group. However, dissimilarity is evident between the four groups, indicating differences among them. For both the bacterial and the plant communities, it was observed that the community structures of the natural and 2009 groups were distinct and clustered separately in the NMDS plot, while the 1989 and 1997 age groups overlapped. The PERMANOVA results indicated that the environmental factors that affect the bacterial community structure in a statistically significant manner include age since abandonment (*p* = 0.001), WHC (*p* = 0.008) and % dead vegetation (*p* = 0.007). For the vegetation community, it was observed that age of abandonment (*p* = 0.002) and WHC (*p* = 0.003), as well as %C (*p* = 0.036), N Stock (*p* = 0.039), C stock (*p* = 0.035) and % dead vegetation (*p* = 0.006) have significant effects on the vegetation community composition. These results suggest that age, soil WHC and % dead vegetation material are the most influential factors affecting both bacterial and plant communities, while carbon and nitrogen stocks also play significant roles in shaping plant community composition.

### 3.3 Differential Abundance Testing

The differential abundance results identified several bacterial ASVs with significant deviations in abundance when comparing the natural group to the 2009 (174 ASVs, *p* < 0.01), 1997 (117 ASVs, *p* < 0.01) and 1989 (126 ASVs, *p* < 0.01) age groups, respectively (Figure S5).

Two bacterial families, Acidobacteriaceae (Subgroup 1; Acidobacteriota) and Planococcaceae (Firmicutes), were consistently more abundant in the natural sites in all three Deseq comparisons. The Acidobacteriaceae genus *Occallatibacter* and the Planococcaceae genera *Sporosarcina* and *Psychrobacillus* were specifically found as significantly more abundant in the natural sites in all comparisons. Four families, Blastocatellaceae (Acidobacteriota), Chitinophagaceae (Bacteroidota), Oxalobacteraceae (Proteobacteria) and Rubrobacteriaceae (Actinobacteria), were observed as more abundant in all three old crop fields. Abundant genera observed in the old crop fields included *Stenotrophobacter* (Blastocatellaceae), *Segetibacter* (Chitinophagaceae), *Massilia* and *Noviherbaspirillum* (Oxalobacteraceae) and *Rubrobacter* (Rubrobacteriaceae).

The vegetation analysis showed that the grass genus *Tenaxia* was more abundant in the natural sites in all three comparisons. The grass genus *Themeda* was differentially more abundant in the natural sites in only the 2009 and 1997 comparisons (Figure S6).

## 4 Discussion

In this study, we assessed the rate of natural recovery for a chronosequence of formerly cultivated fields in the Winterberg mountain range in the Eastern Cape, South Africa. The extent of recovery was examined by considering the composition of bacterial and plant communities, and subsequent diversity levels in the differently aged, abandoned crop fields compared with the surrounding natural habitats. It is important to note that the rarefaction curves generated for the HTS data in this study indicated sufficient sampling depth, although the plant data indicated a trend towards incomplete sampling, suggesting that the observed plant diversity may not be fully representative of the true diversity present in the sampling sites.

There have been varying reports regarding the extent to which old fields can undergo recovery and whether natural biodiversity levels will be attained through techniques such as natural succession. For example, a study by Zhang et al. (2016), carried out in the Loess Plateau of China, reported that bacterial diversity levels were able to recover to the natural diversity levels within 15–20 years. Additionally, a study conducted by Fensham et al. (2016) performed in subtropical grasslands in Queensland, Australia, concluded that grasslands can be restored to their native states, particularly in cases where the grasslands are of ‘natural’ origin rather than being ‘derived’. In contrast to these studies, research by Isbell et al. (2019) in a grassland habitat in Minnesota, USA, showed that old crop fields abandoned for 91 years still only had about three‐quarters of the plant diversity and half of the plant productivity compared with the remnant fields.

No significant differences were observed when comparing Shannon's diversity index and Simpson's diversity index for the bacterial and vegetation communities between the natural sites and the old crop fields. Despite the non‐significant alpha diversity results, a clear trend can still be observed, with the older abandoned fields and natural sites showing higher microbial diversity levels than those observed for the 2009 sites. The inverse is, however, seen for the vegetation data, with the natural sites generally showing lower alpha diversity levels compared with the abandoned crop fields. The wiry tussock grass, *Tenaxia disticha*, is known to dominate the natural grasslands of the Karoo Escarpment Grassland, with a notably low shrub component (Mucina and Rutherford 2006), explaining the lower species richness observed in our natural sites. The abundance of *T. disticha* in the natural sites could further be explained by the land‐use management practices employed at the study site. Controlled burning is not regularly performed (J.M. Coetzer, personal communication), which has been shown to increase the abundance of *T. disticha* and decrease the abundance of more palatable grass species such as *Themeda triandra* and *Heteropogon contortus* (Munyai et al. 2023). The community composition results identified clear variations in both the bacterial and plant community compositions among the four age groups. These results suggest that the overall diversity within the four age groups remains relatively stable as the sites recover over time, while the abundance and distribution of the observed species still vary significantly between the groups. These observations correlate with a study by Yan et al. (2020), which found that sites revegetated between 11 and 15 years before sampling had similar bacterial communities associated with them, while being distinct from the older revegetated sites (16 years and older) and remnant sites. The effect of environmental factors was tested using PERMANOVA, revealing that age since abandonment, the WHC of the soil, as well as the presence of dead vegetation in the older fields and natural habitats, had statistically significant effects on the bacterial and plant communities. It was further observed that % C, N stock (tN/ha), and C stock (tN/ha) have significant effects on plant community composition. Coetzer and Coetzer (2023) investigated the soil quality at the same locations as the present study and reported that the WHC, soil carbon and nitrogen percentages significantly increase with the successional age of the old crop fields. The current investigation did not uncover any significant effects of nitrogen or carbon content on the overall bacterial communities. Despite the lack of significant results in the present research, it is noteworthy that existing literature commonly reports these nutrients as influential factors on these communities (Stone et al. 2021; Jing et al. 2022; Hartmann and Six 2023).

In the current study, the taxa of the different bacterial communities were analysed to explore the dynamics of the dominant phyla along the chronosequence. The results indicate that the top bacterial phyla, Proteobacteria, Actinobacteria, Gemmatimonadetes, Firmicutes and Planctomycetes, were present in all bacterial communities. The soils in all four age groups were dominated by Actinobacteria and Proteobacteria. These results align with previous studies where it was found that these two bacterial taxa are known to dominate various soil types (Zhang et al. 2016; Dube et al. 2019; Cowan et al. 2022). The observed trends in bacterial phylum abundances from the present study highlight the effect of study site on bacterial abundances, with contrasting observations reported in literature (Zhang et al. 2016; Silva et al. 2024). Following the old‐field succession, the relative abundance of Proteobacteria steadily increased with an increase in time since abandonment. Several studies, including research by Zhang et al. (2012) and Zhang et al. (2016) in the Loess Plateau in China, reported bacterial communities steadily transitioning from Actinobacteria‐dominant to Proteobacteria‐dominant communities during successional periods. Additionally, a study by Dube et al. (2019), performed in the Free State Province, South Africa, found that agricultural land use shifted soils from being oligotrophic (nutrient‐poor) to copiotrophic (nutrient‐rich), which changed bacterial communities from being Actinobacteria‐dominated to Proteobacteria‐dominated. Proteobacteria are known to thrive in nutrient‐rich—high C availability—soils, and are linked to a preference for organic matter decomposition (Fierer et al. 2011; Wang et al. 2019), supporting the relatively higher abundance observed in the natural and 1989 sites. It has been well documented that vegetation restoration can increase the relative abundance of Proteobacteria due to the positive impact that soil organic carbon has on the survival and growth of this bacterial phylum (Hartman et al. 2008; Zeng et al. 2017). The associations between Proteobacteria and soil nutrient content are noteworthy, as members of this phylum are known for their diverse metabolic capabilities (Spain et al. 2009). Silva et al. (2024), however, observed increased levels of Proteobacteria in degraded environments in a Brazilian semi‐arid region, highlighting the contrasting observations found in different study sites.

Interestingly, we observed no significant trends for Actinobacteria in the present study. It has been reported that Actinobacteria play an important ecophysiological role in plant matter decomposition and show a stable presence during plant decomposition (Bao et al. 2021). The relatively stable presence across sites observed in our study could be explained by the role in plant decomposition, as the natural sites had high amounts of dead/decaying plant material. Actinobacteria also show functional importance in less fertile soils (Bao et al. 2021), which further explains the presence in the younger abandoned crop fields. Our observations of the five bacterial phyla associated with the younger abandoned crop fields are also well supported in literature. Chloroflexi have been reported at higher abundance levels in diverse environments, including degraded Brazilian semi‐arid soils (Silva et al. 2024), and managed forests and crop lands in Southwest China (Lan et al. 2022). Chloroflexi, Nitrospirota and Planctomycetota were enriched in croplands and fallow soils globally (Bai et al. 2022; Lavrishchev et al. 2024; Peng et al. 2024). Chloroflexi, Abditibacteriota and Armatimonadota showed significant negative correlations to total N in a study by Chen et al. (2025), supporting our observations of higher abundances in the younger abandoned crop fields, which showed lower N levels (Coetzer and Coetzer 2023). The observed shifts in the abundance of these bacterial phyla could indicate successional changes, reflecting the complex ecological responses of these microorganisms during natural recovery.

The family level differential abundance tests showed a slightly different picture, with taxa from Acidobacteriaceae (Acidobacteriota) and Planococcaceae (Firmicutes) being more abundant in the natural sites. These families are known to contribute to soil nutrient cycling and organic matter decomposition (Campbell 2014; Shivaji et al. 2014; Dedysh and Damsté 2018). The large amount of dead plant material, in the form of moribund, observed in the natural sites most probably provides sufficient organic matter to stimulate the development of Acidobacteriaceae and Planococcaceae colonies. This also supports the PERMANOVA findings from the NMDS analyses, identifying the significant role dead vegetation plays in the microbial community structure. Members of the Acidobacteriaceae family are known to tolerate acidic conditions. They are found in a vast array of habitats including acidic soils, peat bogs and acidic mine waste (Campbell 2014; Dedysh and Damsté 2018). The Acidobacteriaceae are known to produce exopolysaccharides (EPS) which presumably assist bacteria in surviving environmental stressors such as high acidity and low temperatures, and also contribute to soil moisture retention (Dedysh and Damsté 2018; Bhagat et al. 2021). The presence of these bacteria could help explain the higher WHC observed for the soils at the natural sites (Coetzer and Coetzer 2023). Planococcaceae taxa such as *Sporosarcina* and *Psychrobacillus* are known for their roles in nutrient cycling, specifically nitrogen cycling and phosphate solubilisation (Chiba et al. 2022; Jhuo et al. 2025), highlighting their potential role in soil fertility and plant growth stimulation.

The four bacterial families (Blastocatellaceae, Rubrobacteriaceae, Oxalobacteraceae and Chitinophagaceae) identified as the most abundant in the old crop fields are all known for their ability to grow in nutrient‐poor soils, resist stressful conditions, contribute to nitrogen and carbon cycling, and thereby possibly support plant health in recovering ecosystems. The Blastocatellaceae family (Phylum Actinobacteria) includes genera such as *Aridibacter*, *Blastocatella* and *Stenotrophobacter* and is typically found in soil environments. These bacteria are known for their ability to thrive in nutrient‐poor conditions. They are chemoheterotrophic, meaning they obtain their energy from the oxidation of organic compounds (Pascual et al. 2015). Blastocatellaceae bacterial taxa are known to survive in extreme environments and have been isolated from semi‐arid savanna soils in Namibia (Pascual et al. 2015; Wüst et al. 2016). The Rubrobacteriaceae family (Phylum Actinobacteria) includes the genus *Rubrobacter*, which is known for its extreme resistance to ionising radiation and desiccation. These bacteria are typically found in hot environments and are moderately thermophilic (Albuquerque and da Costa 2014). Members of this family have previously been identified in sandy clay rangeland soils and desert soils in Australia (Holmes et al. 2000; Vega‐Cofre et al. 2023), and can survive nutrient‐poor conditions (Chen et al. 2022). They contribute to the degradation of organic materials in extreme environments and play important roles in carbon, nitrogen and sulphur cycling (Chen et al. 2022). The Oxalobacteraceae family (Phylum Proteobacteria) includes genera such as *Oxalobacter*, *Herbaspirillum* and *Janthinobacterium*, which exhibit a wide range of metabolic capabilities, including nitrogen fixation and the degradation of oxalate and chitin (Chen et al. 2023). Members from this family can be found in diverse environments, including soil, water and plant‐associated habitats (Baldani et al. 2014), and are regularly associated with the plant rhizosphere, involved in nutrient cycling (Ofek et al. 2012). Members of the family Chitinophagaceae (Phylum Bacteroidota) are known to degrade cellulose and chitin, which are major components of plant and fungal cellular structures (Veliz et al. 2017; Chen et al. 2023; Huang et al. 2023). Taxa from this family have been previously observed in both actively farmed olive groves and abandoned groves, with lower numbers observed in abandoned olive groves (Company et al. 2022). Research by Khan et al. (2023) indicated a higher abundance of Chitinophagaceae in fields following a no‐till approach, and the Chitinophagaceae genus *Segetibacter* has previously been identified as an indicator taxon for natural soils in a semi‐arid region in Spain (Rodríguez‐Berbel et al. 2023). The fact that members of Chitinophagaceae are generally observed in high biological materials such as plant and/or fungal matter could indicate that the old crop fields are indeed in an advanced stage of recovery.

The upregulation of these families in the different stages of recovery suggests an adaptive response, possibly linked to the differences in soil conditions and plant community structures. Further investigation is needed to understand the biological significance of these upregulations and the potential implications for the studied system. In the present study, bare ground observations decreased with successional age, whereas dead vegetation (e.g., moribund) was mainly found in the natural sites, with negligibly low numbers observed in the old crop fields. Dead vegetation plays a dual role by increasing soil nutrient content and improving WHC by binding soil particles into aggregates (Mohammadi et al. 2011). Additionally, WHC has been associated with improved moisture conditions, creating more favourable conditions for bacterial growth and activity (Sun et al. 2015; Huang et al. 2019).

The presence and characteristics of vegetation communities additionally influence the complex relationships between bacterial populations and soil properties. The plant communities in the current study are dominated by the family Poaceae, aligning with the expected vegetation found within temperate grassland biomes (Carbutt et al. 2011; Carbutt and Kirkman 2022). The vegetation in the 2009 sites exhibits characteristics of being in a transitional stage between the pioneer and subclimax stages. The main plant species in these sites are predominantly species from pioneer genera including *Eragrostis* and *Cynodon*, but the presence of subclimax species indicates an ongoing progression from the pioneer to the subclimax stage. Additionally, the 1997 and 1989 sites can be classified as being in the subclimax stage, where the pioneer species have effectively transformed the environment, allowing the establishment of perennial grass species. The subclimax grasses could eventually give way to climax grasses, with growth conditions continuing to improve. Species from the genera *Eragrostis* and *Melica* dominate both groups (1997 and 1989). In terms of relative abundance, the 1997 group shows a higher representation of the *Helictotrichon* genus, while the 1989 group exhibits a higher representation of the *Tenaxia* genus. This disparity suggests that the 1989 group is closer to transitioning to climax grasses, whereas the 1997 group is still in an earlier stage of succession. However, given the drastic climate and rainfall shifts in the area, it is uncertain whether the old fields will progress beyond the subclimax stage, preventing the complete restoration of the old fields to natural conditions (van Oudtshoorn 2015, 2020). It is important to note that potential contributors to the differences in the plant community compositions in this study include grazing and trampling by livestock. It has been reported that livestock grazing could restrict the accumulation of grazing‐sensitive perennial grass and forb species (Fensham et al. 2016). Given that both the abandoned croplands and natural habitats in the current study are exposed to livestock grazing, it is conceivable that species favoured by grazing animals may be infrequent or absent, while other species less preferred by grazing may exhibit a higher abundance. These plant communities significantly shape microbial communities (Wardle et al. 2004). Thus, the significant differences observed between the plant community compositions may affect the associated bacterial community compositions, as different plant species can have distinct effects on various ecosystem processes (Wardle et al. 2004; Allan et al. 2013).

There are multiple potential reasons for the observed results regarding the abundance and distribution of these bacterial families, including soil moisture (DeBruyn Jennifer et al. 2011), nutrient availability (Delgado‐Baquerizo et al. 2017) and pH levels (Kang et al. 2021). Specifically, the current study demonstrates associations between the vegetation and specific bacterial families, as well as significant impacts of dead vegetation and WHC on bacterial community compositions. Understanding these intricate relationships allows for a better understanding of soil health and how it affects ecosystem functioning; thus, further research is needed to thoroughly investigate and understand the effect these factors have on the soil microbiome, especially in South African grasslands.

## 5 Conclusion

Biodiversity analyses, including the one presented in this study, are essential to evaluate the impact of human activity in an ecosystem. Measuring plant and microbial diversity in abandoned crop fields in different successional stages will increase the knowledge of the diversity of natural ecosystems versus modified ones and, ultimately, the rate at which recovery can be achieved through remedial actions such as abandonment. Studying these communities will allow researchers to identify the patterns associated with ecosystem recovery, which can help society regulate and conserve biodiversity, especially within the agricultural sector. The biodiversity levels of the old fields have recovered to a point where they resemble natural conditions; however, given the substantial climate changes in the area, it remains uncertain whether the old fields will reach native conditions in the coming decades, if at all. Something to consider for future studies is to increase the sampling effort during the vegetation surveys; this would ensure a more comprehensive representation of the vegetation community during downstream analyses. Further investigation into the biological significance of the observed bacterial family upregulations is also crucial for a more complete understanding of ecosystem functioning in these recovering landscapes.

## Author Contributions

**Heike Oosthuysen:** data curation (equal), formal analysis (lead), investigation (equal), validation (supporting), visualization (lead), writing – original draft (lead), writing – review and editing (equal). **Kayleigh Coetzer:** conceptualization (equal), methodology (supporting), supervision (supporting), validation (equal), writing – review and editing (equal). **M. Thabang Madisha:** conceptualization (supporting), funding acquisition (supporting), methodology (equal), supervision (supporting), validation (supporting), writing – review and editing (equal). **Willem G. Coetzer:** conceptualization (lead), formal analysis (supporting), funding acquisition (lead), investigation (supporting), project administration (lead), resources (lead), supervision (lead), validation (equal), visualization (supporting), writing – review and editing (equal).

## Funding

This work was supported by the Universiteit van die Vrystaat, the 50% special projects: central research fund (C).

## Conflicts of Interest

The authors declare no conflicts of interest.

## Supporting information


**Figures S1–S6:** ece372865‐sup‐0001‐FiguresS1‐S6.docx.

**Table S1:** ece372865‐sup‐0002‐TableS1.xlsx.

**Table S2:** ece372865‐sup‐0003‐TableS2.docx.

## Data Availability

All raw data are submitted to GenBank under BioProject PRJNA1043901. See Table 1 for details on accession numbers.
