# Supplementary material for: An Analysis of Vegetation and Microbiome Recovery in Abandoned Agricultural Fields
Source: Ecol Evol. 2026 Jan 5;16(1):e72865. doi: 10.1002/ece3.72865 (PMC12771683; doi:10.1002/ece3.72865)
Supplement: Supplementary file 1 — Figures S1–S6: ece372865‐sup‐0001‐FiguresS1‐S6.docx. [file ECE3-16-e72865-s002.docx]

**Supplementary Figures:** An analysis of vegetation and microbiome recovery in abandoned agricultural fields.

**Authors and affiliations:**

Heike Oosthuysen ^1^, Kayleigh Coetzer ^2^, M. Thabang Madisha ^1^ and Willem G. Coetzer ^3,^ *

^1^ Department of Genetics, University of the Free State, Bloemfontein, Free State, South Africa

^2^ Leliekloof Farm, Cradock, Eastern Cape, South Africa

^3^ Department of Zoology and Entomology, University of Fort Hare, Eastern Cape, South Africa

*** Corresponding Author:**

Willem G. Coetzer (coetzerwg@outlook.com), Department of Zoology and Entomology, University of Fort Hare, Eastern Cape, South Africa


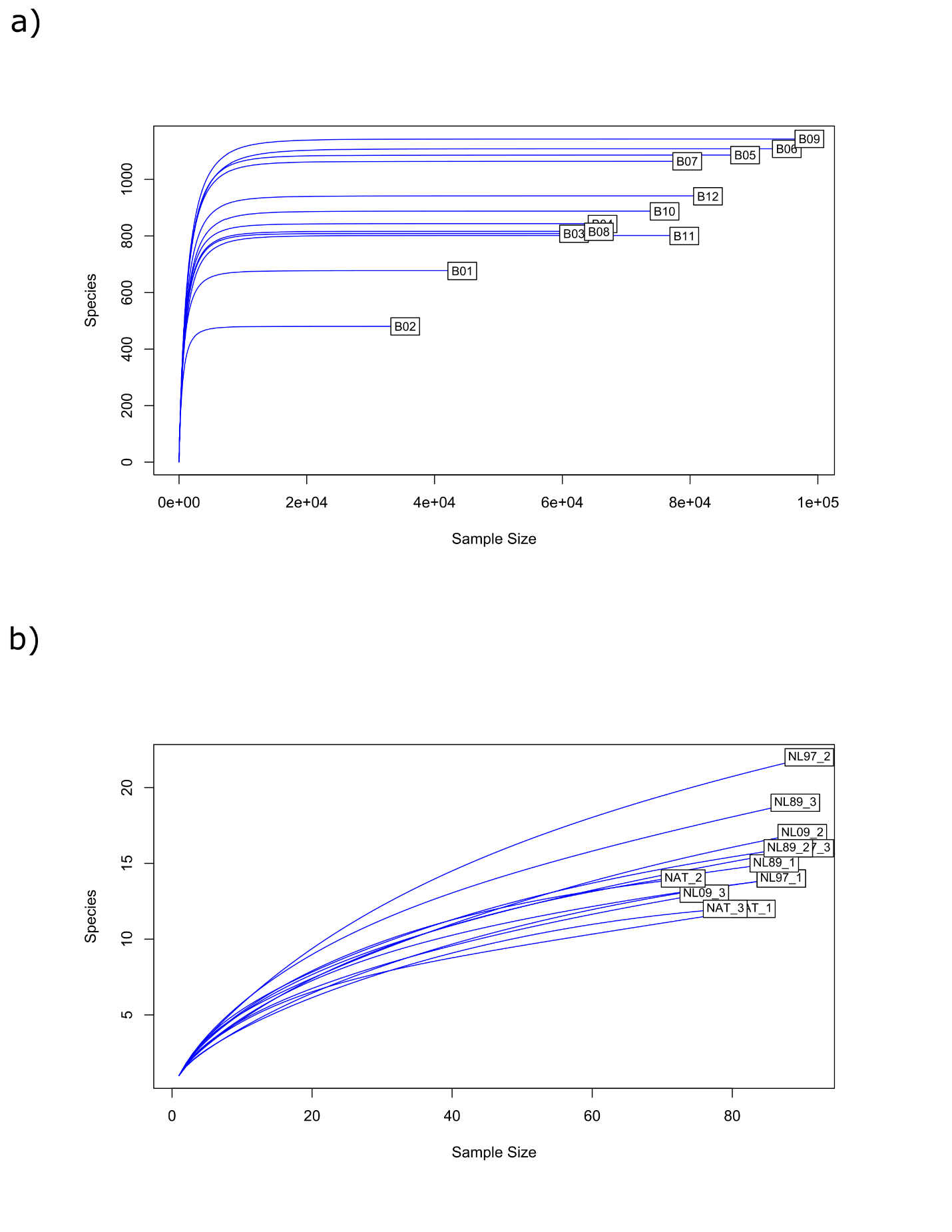


**Figure S1.** Sample accumulation curves for a) the microbiome data and b) the vegetation count data.

**
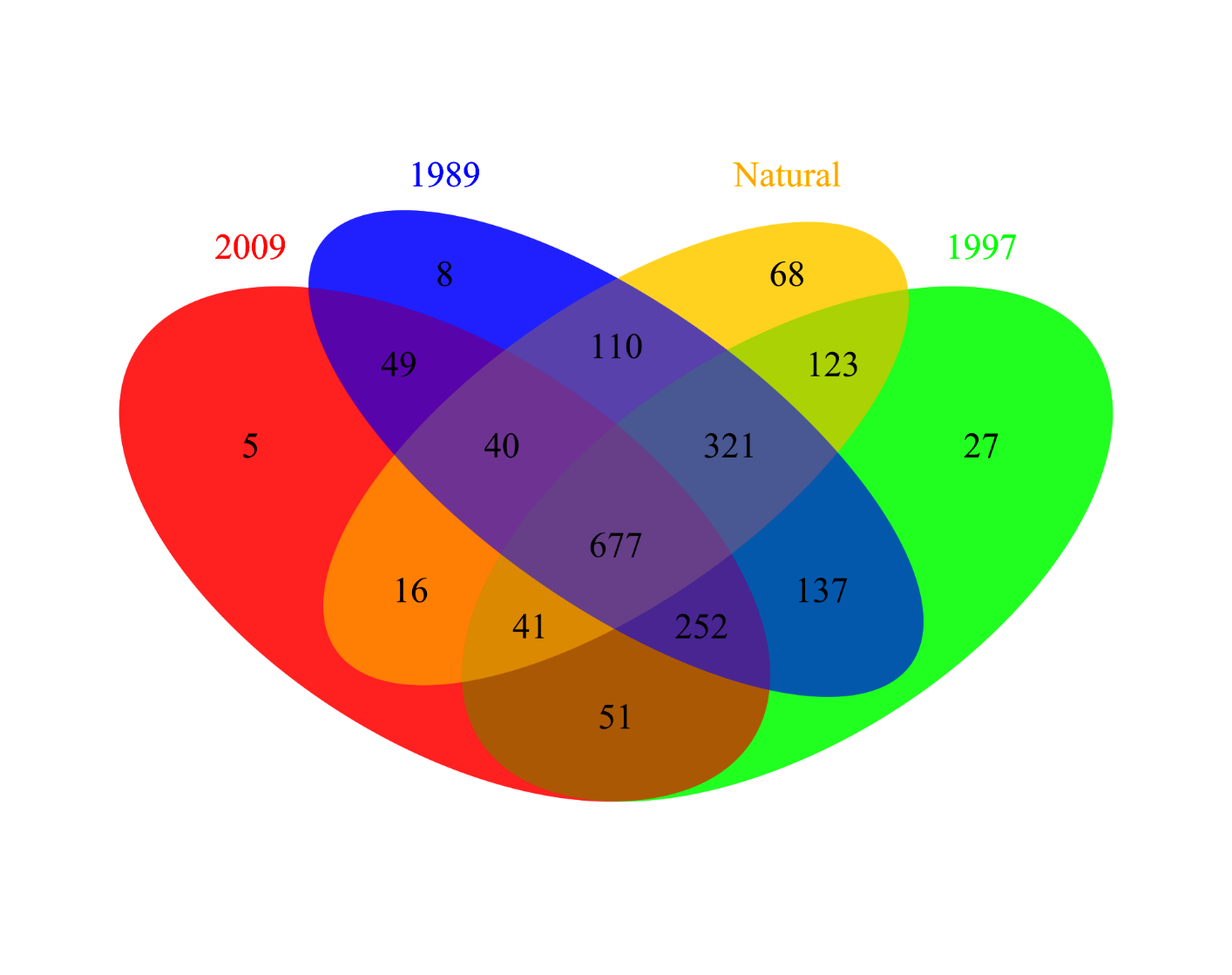
**

**Figure S2.** A Venn diagram visualizing the bacterial ASV distribution across the different sample sites. It can be observed that 677 ASVs are shared by all sites.


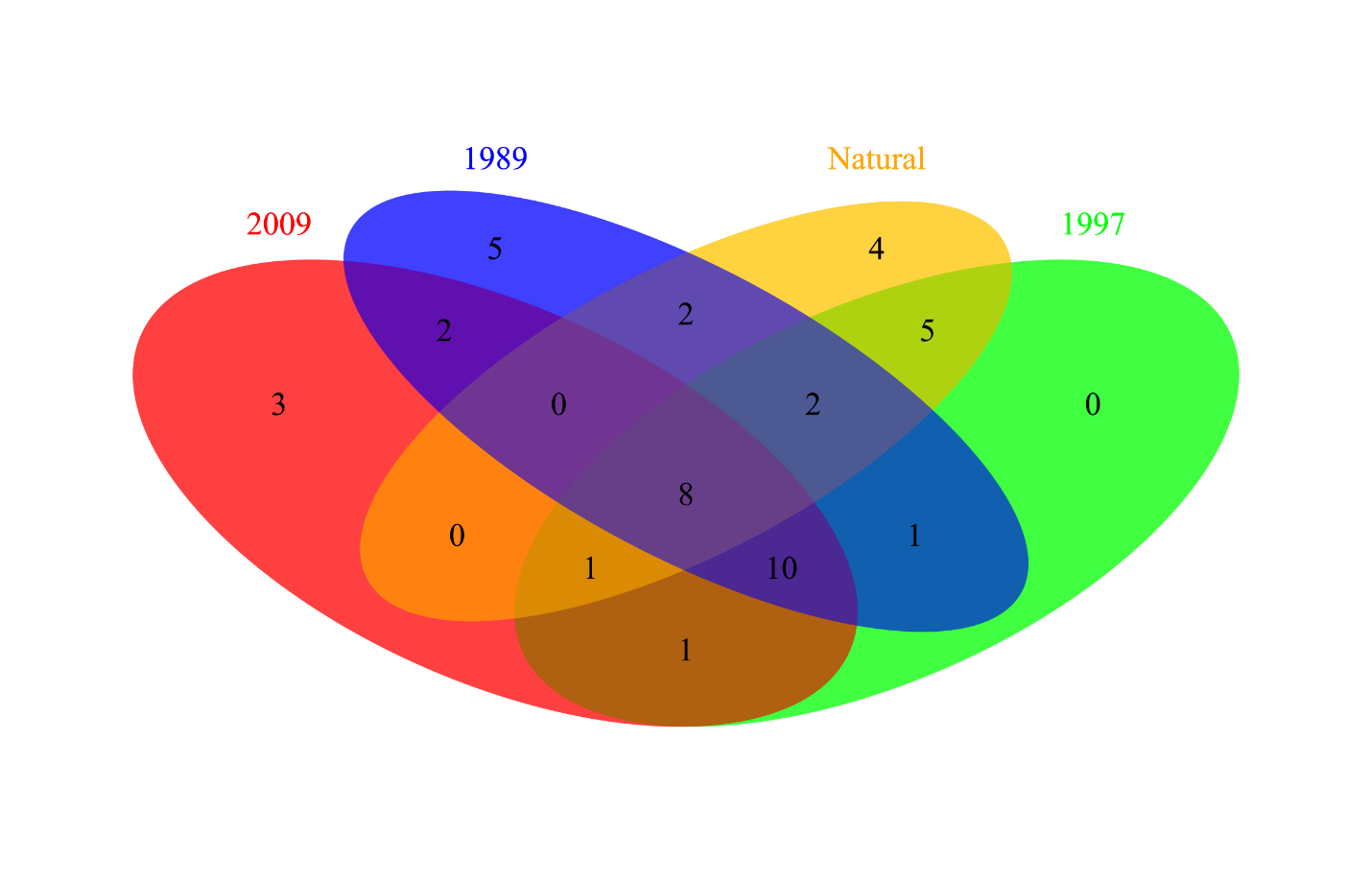


**Figure S3.** A Venn diagram visualizing the vegetation species distribution across the different sample sites. It can be observed that 8 species are shared by all sites.


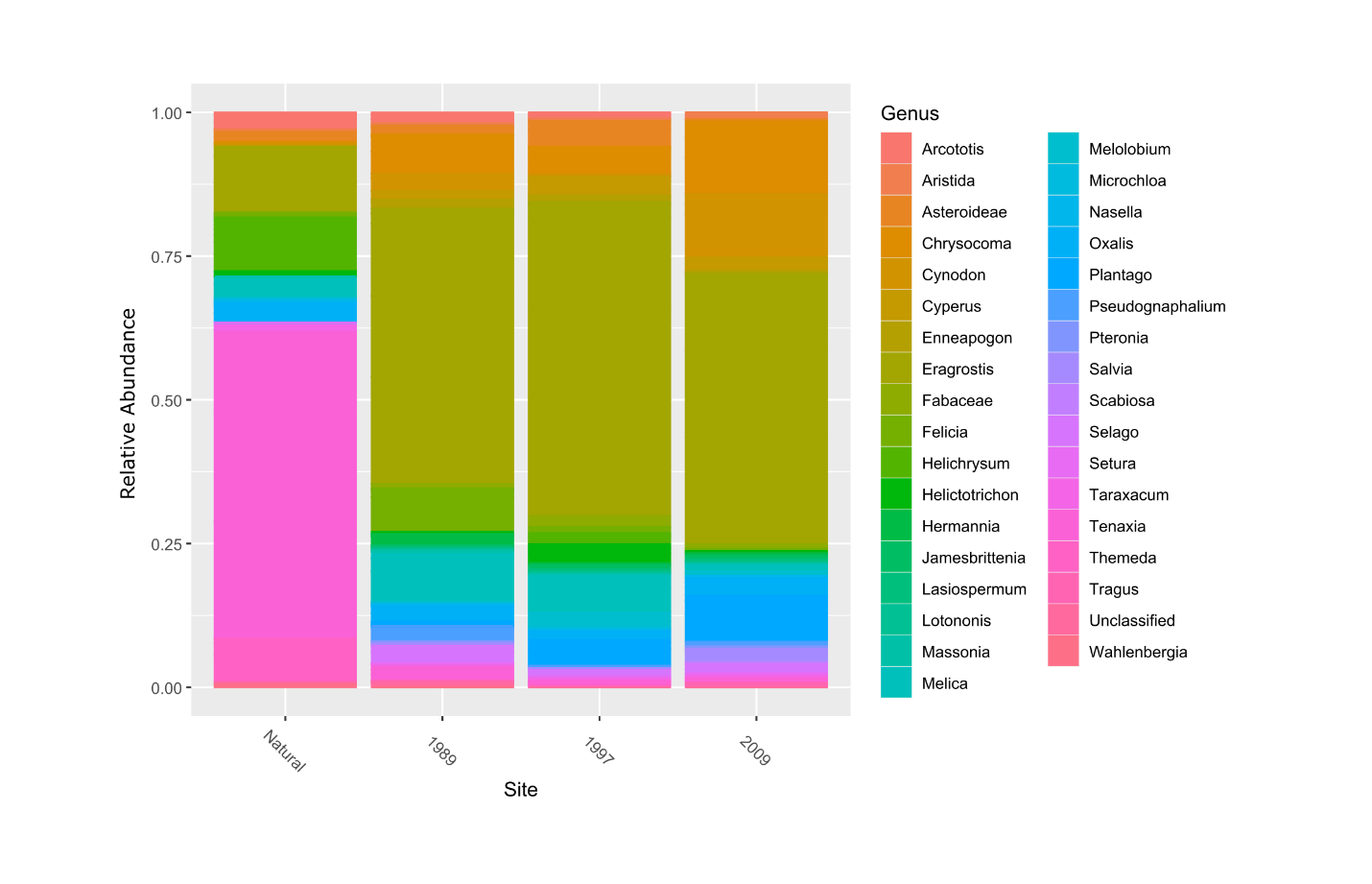


**Figures S4.** The genus level Relative abundance plot for the vegetation data, pooled per site.


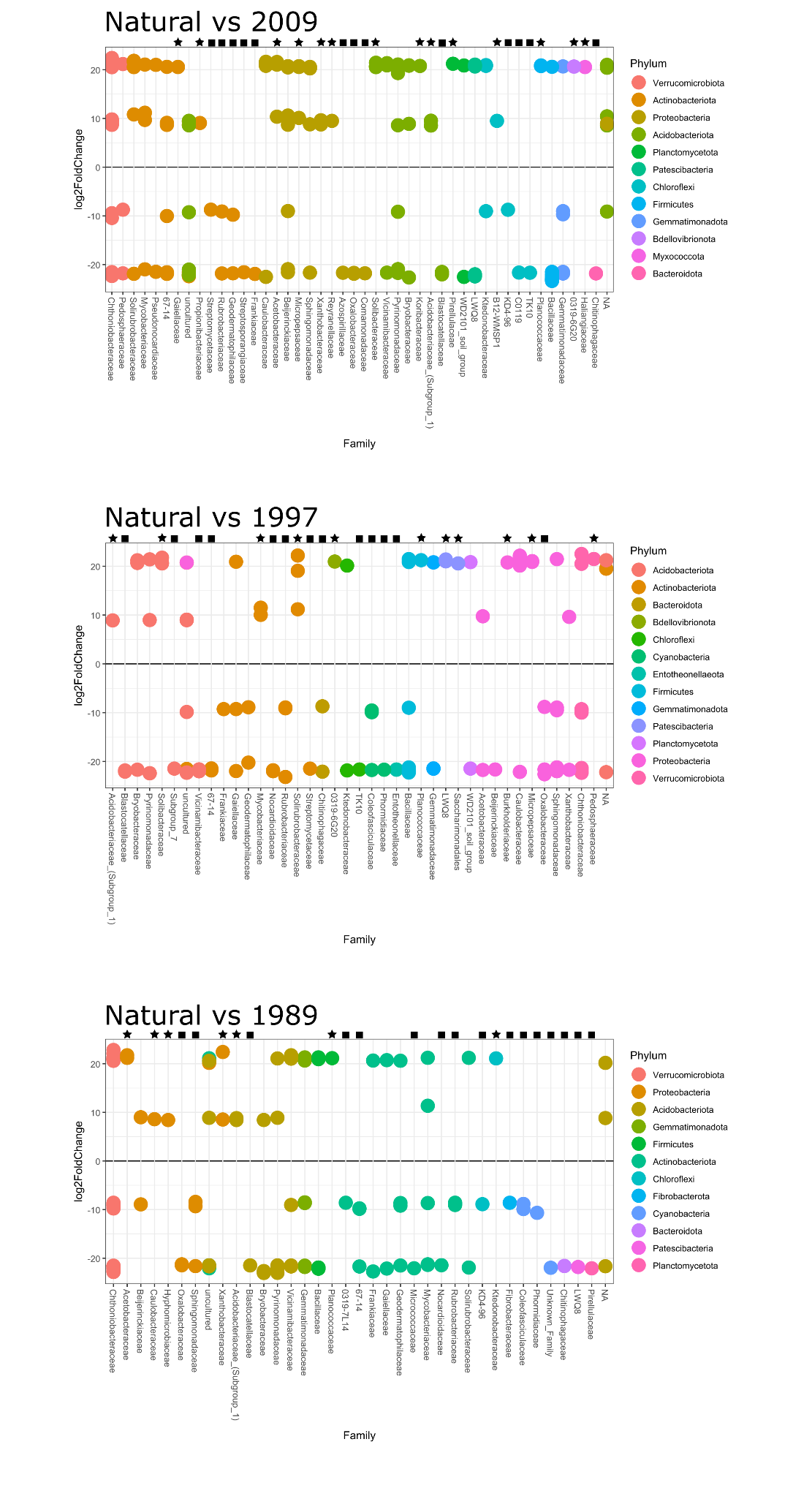


**Figure S5.** The Deseq2 results visualizing the bacterial family level differential abundance assessments for the natural sites compared to each of the old crop fields. Positive log2FoldChange values indicate increased abundance in the natural sites (control) and negative values allude to increased abundance in the old crop fields.

**
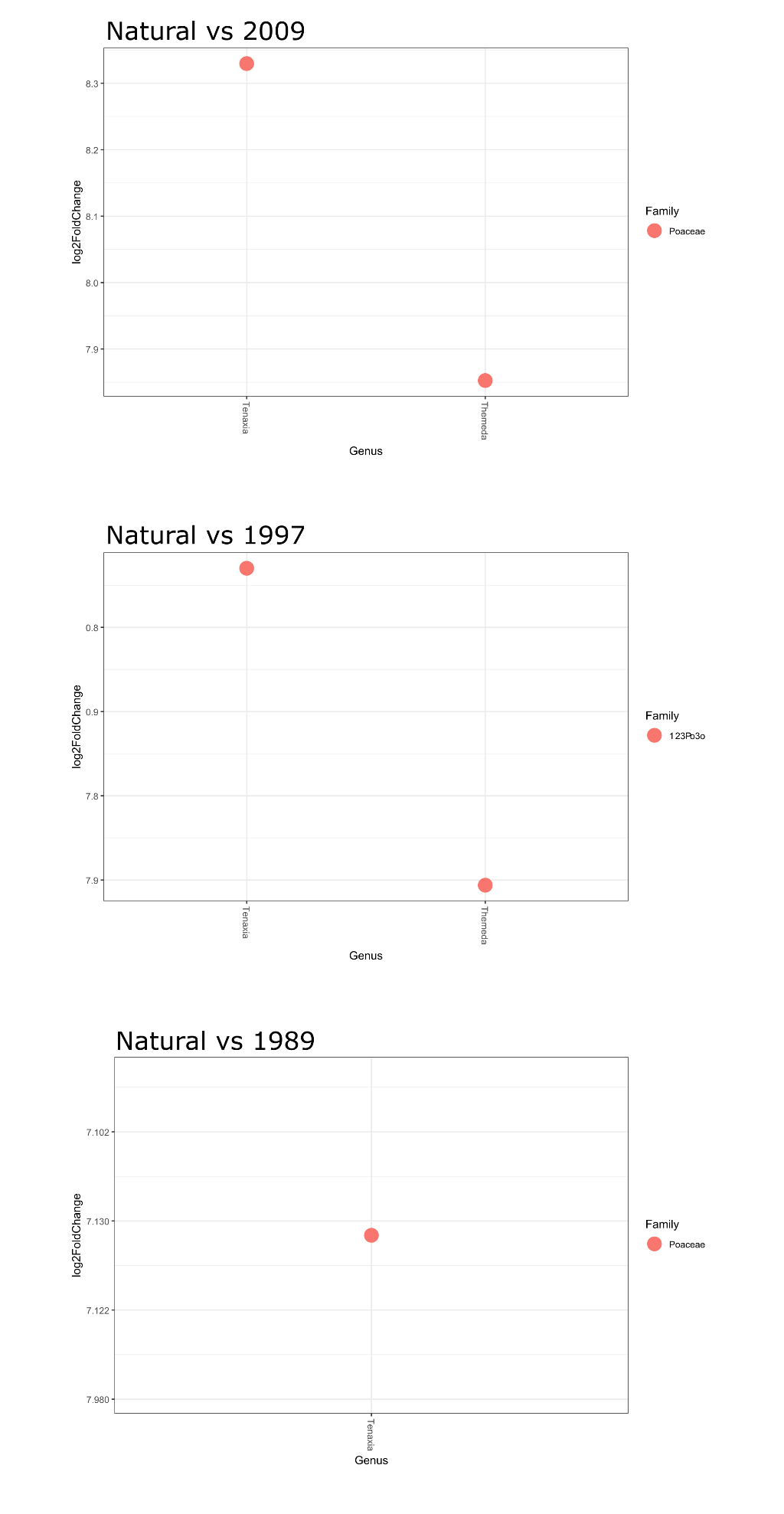
**

**Figure S6.** The Deseq2 results visualizing the vegetation genus level differential abundance assessments for the natural sites compared to each of the old crop fields. Positive log2FoldChange values indicate increased abundance in the natural sites (control) and negative values allude to increased abundance in the old crop fields.
