# Supplementary material for: An Analysis of Vegetation and Microbiome Recovery in Abandoned Agricultural Fields
Source: Ecol Evol. 2026 Jan 5;16(1):e72865. doi: 10.1002/ece3.72865 (PMC12771683; doi:10.1002/ece3.72865)
Supplement: Supplementary file 3 — Table S2: ece372865‐sup‐0003‐TableS2.docx. [file ECE3-16-e72865-s001.docx]

**Supplementary Table S2.** Below we summarize the top three taxa for a) the bacterial abundances at Phylum and Family level, and b) the vegetation abundances at the Family and Genus levels.

1. The top three bacterial Phyla and Families for the site groupings.

| Group | Top 3 Phyla | Top 3 Families |
| --- | --- | --- |
| 2009 Group | Actinobacteriota: 24.20%, Proteobacteria: 21.17%, Acidobacteriota: 16.21% | Chthoniobacteraceae: 15.42%, uncultured: 10.28%, Gemmatimonadaceae: 7.76% |
| 1997 Group | Proteobacteria: 25.66%, Actinobacteriota: 22.56%, Acidobacteriota: 17.96% | Chthoniobacteraceae: 14.94%, uncultured: 9.96%, Xanthobacteraceae: 7.56% |
| 1989 Group | Proteobacteria: 26.88%, Actinobacteriota: 24.99%, Verrucomicrobiota: 14.92% | Chthoniobacteraceae: 14.94%, uncultured: 9.96%, Xanthobacteraceae: 7.56% |
| Natural Group | Proteobacteria: 31.08%, Actinobacteriota: 22.70%, Acidobacteriota: 18.76% | Chthoniobacteraceae: 13.94%, uncultured: 10.83%, Xanthobacteraceae: 10.49% |

1. The top three plant Families and Genera for the site groupings.

| Group | Top 3 Families | Top 3 Genera |
| --- | --- | --- |
| 2009 group | Poaceae: 63.2%,  Asteraceae: 15.4%, Plantaginaceae: 8.3% | Eragrostis: 47.0%, Chrysocoma: 13.0%, Cynodon: 11.1% |
| 1997 group | Poaceae: 67.7%,  Asteraceae: 14.5%,  Fabaceae: 4.8% | Eragrostis: 54.6%,  Melica: 6.7%,  Asteroideae: 4.8% |
| 1989 group | Poaceae: 64.6%,  Asteraceae: 21.3%, Scrophulariaceae: 3.4% | Eragrostis: 47.9%,  Melica: 8.7%,  Felicia: 7.6% |
| Natural group | Poaceae: 77.9%,  Asteraceae: 16.6%,  Oxalidaceae: 3.8% | Tenaxia: 53.6%,  Eragrostis: 11.5%, Helichrysum: 9.4% |
